# Predicting the Perceived Sound Quality of Frequency-Compressed Speech

**DOI:** 10.1371/journal.pone.0110260

**Published:** 2014-11-17

**Authors:** Rainer Huber, Vijay Parsa, Susan Scollie

**Affiliations:** 1 Centre of Competence HörTech gGmbH, Oldenburg, Germany; 2 Cluster of Excellence Hearing4All, Oldenburg and Hannover, Germany; 3 National Centre for Audiology, Western University, London, Canada; University of South Florida, United States of America

## Abstract

The performance of objective speech and audio quality measures for the prediction of the perceived quality of frequency-compressed speech in hearing aids is investigated in this paper. A number of existing quality measures have been applied to speech signals processed by a hearing aid, which compresses speech spectra along frequency in order to make information contained in higher frequencies audible for listeners with severe high-frequency hearing loss. Quality measures were compared with subjective ratings obtained from normal hearing and hearing impaired children and adults in an earlier study. High correlations were achieved with quality measures computed by quality models that are based on the auditory model of Dau et al., namely, the measure PSM, computed by the quality model PEMO-Q; the measure qc, computed by the quality model proposed by Hansen and Kollmeier; and the linear subcomponent of the HASQI. For the prediction of quality ratings by hearing impaired listeners, extensions of some models incorporating hearing loss were implemented and shown to achieve improved prediction accuracy. Results indicate that these objective quality measures can potentially serve as tools for assisting in initial setting of frequency compression parameters.

## Introduction

### Nonlinear frequency compression

Frequency lowering techniques are now common in digital hearing aids as an alternative amplification strategy for hearing impaired listeners with severe to profound high frequency hearing loss. For this group of listeners, conventional amplification strategies may result in less than optimal performance due to a combination of inadequate gain at higher frequencies, limited bandwidth of the hearing instruments, and/or the potential presence of high frequency cochlear dead regions [1], [2], among other factors. Frequency lowering techniques aim to transfer high frequency information to lower frequency regions. These techniques can improve speech recognition, but may also affect perceived sound quality. In this paper, we investigate a broad range of objective indices of sound quality against a database of sound quality ratings for frequency-lowered speech. This work not only evaluates which models may be effective predictors of the impact of one form of frequency lowering on sound quality, but also develops insights as to the key features of a successful model by varying key modelling parameters and evaluating their impact on successful predictions.

Historically, frequency lowering has been achieved in many ways including slow playback, channel vocoding, frequency transposition, and frequency compression, as well as a combination of these alternative strategies [3]. As described in ([3] - Section 8.3), a frequency transposing hearing aid will shift a portion of high frequency spectrum to lower frequencies by a fixed amount (in Hz). Frequency compression can be linear or nonlinear; in linear frequency compression, all frequencies are proportionally reduced by the same factor (i.e., the output frequency is a fixed fraction of the input frequency), while in nonlinear frequency compression, only frequencies above a certain threshold are compressed.

Currently, frequency lowering is available as an option in commercial hearing aids as frequency transposition (termed “Audibility Extender”) in Widex hearing aids [4], as nonlinear frequency compression (termed “SoundRecover”) in Phonak and Unitron hearing aids and as “frequency compression” in Siemens hearing aids, as spectral warping (termed “SpectralIQ”) in Starkey hearing aids, and as “Frequency Composition”, another type of frequency transposition [5], in Bernafon hearing aids.

The differences in frequency lowering implementations can be expected to result in considerably different perceptual effects, even when they are fitted for the same audiometric configuration. For example, McDermott [6] conducted an electroacoustic comparison of Widex’s Audibility Extender and Phonak’s Sound Recover technologies. Spectrographic analyses with different speech and music samples showed that while both schemes were effective in lowering high frequency content, they also introduced distortion that may affect perception. It is therefore imperative to investigate the perceptual effects of frequency lowering technologies, and to develop electroacoustic tools and computational models that can predict these perceptual effects.

Since this paper focuses on a particular frequency lowering strategy, *viz.* the nonlinear frequency compression (NFC), a brief description of the NFC processing and the evidence surrounding its effectiveness is presented below.

**Figure 1:**
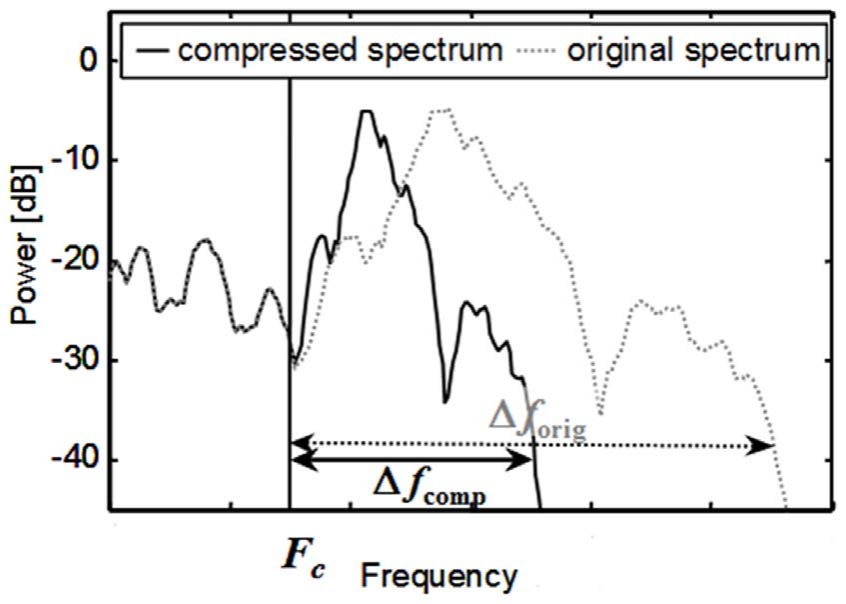
Illustration of nonlinear frequency compression. Dotted, grey line: Power spectrum of unprocessed speech. Solid, black line: spectrum after nonlinear frequency compression. Above a cutoff frequency *F_c_,* the spectrum is compressed by a ratio CR = Δ*f*_orig_/Δ*f*_comp_.

The NFC processing in today’s commercial hearing aids uses compression only for frequencies above a cutoff value. Mathematically, the relationship between input and output frequencies is given by
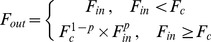
(1)where *F_in_* is the input frequency in Hz, *F_out_* is the corresponding output frequency, *F_c_* is the cutoff frequency, *p* is the compression exponent, and *1/p* is the compression ratio (CR). Figure 1 illustrates the concept showing short time spectra of speech before and after nonlinear frequency compression.

The specification of the *F_c_* ensures that the lower frequency information is unadulterated while the spectral content beyond the *F_c_* is compressed into a narrower bandwidth. The NFC scheme has been evaluated with both adults and children. Simpson *et al.* [7] investigated the performance of prototype NFC with 17 hearing impaired adults with moderate to profound sensorineural hearing loss and found that the phoneme recognition scores for the group improved by 6%. Glista *et al.* [8] evaluated NFC processing with 13 adults and 11 children with moderately severe to profound sloping high-frequency hearing losses. On average, recognition scores for plurals and consonants improved significantly for the NFC scheme when compared with conventional amplification. Individual variability was present in the results and children derived greater plural recognition benefit from NFC as well as indicated preference for NFC over conventional amplification when compared to the adults [8]. Benefit was also related to audiogram: Those with greater high frequency hearing loss were most likely to demonstrate benefits. More recent studies have evaluated outcomes for those with moderate hearing losses [9], the time course of acclimatization [10] and effects on sound quality [11], [12]. Previous work indicated that sound quality is correlated with the strength of the frequency compressor, with stronger settings having poorer sound quality. This underscores the need for formal evaluation of sound quality of the NFC processor. In particular, computational metrics which can effectively predict speech quality perception by hearing impaired listeners could be of use in determining an initial set of NFC parameters that have acceptable sound quality, potentially improving the acceptability of initial fittings.

### Objective sound quality evaluation

The perceived quality of frequency-compressed sounds will depend on a number of variables, including the parameter settings, hearing loss, the type of sound, as well as highly individual, subjective factors like personal experiences, expectations and preferences. In order to estimate the expected sound quality for a given algorithm setting, sound, and listener, one could either follow a data-driven, i.e. statistical model approach (e.g, the E-model [13]), or a perception model approach (e.g. PESQ [14]). A data-driven approach would require a large base of empirical data containing subjective quality ratings of different types of sounds, processed with a variety of algorithm settings, obtained from many listeners with different hearing losses. A perception-model-driven approach would possibly require similar amounts of data if it was developed, trained or optimized, and validated particularly for this application.

Alternatively, existing quality models already validated for similar applications could be tested with a smaller amount of data. However, most of the existing quality models were designed, optimized and validated in the context of telecommunication applications and audio coding for normal-hearing listeners (see [15] for an overview). At present, very few sound quality models for hearing impaired listeners with application for hearing aid (algorithm) quality evaluations have been reported [16]–[19]. Most of the sound quality models, including current ITU standard methods for speech and audio quality, have in common that they follow the concept of comparing “internal representations”, computed by a psychoacoustic model, of a test and a reference sound signal [20], [14]. Detected differences between internal representations are interpreted as quality degradations of the test signal with respect to the reference signal. Hence, these comparison-based models depend on the availability of a reference signal that represents the optimum, or desired, sound quality. This requirement is met in the evaluation of lossy signal processing systems, such as low-bitrate speech and audio codecs, where the unprocessed, original signal serves as a reference.

However, there is no known ideal reference for the evaluation of hearing aids (algorithms) in general. In contrast to audio codecs, whose aim is to produce output signals that are perceptually indistinguishable from the original input, hearing aids aim to alter the input sound in a way that it compensates for the listeners’ hearing loss. This intentional alternation could result in the processed sound having higher sound quality than the original signal. Therefore, one would need a “perfect” hearing aid to produce the reference signal for sound quality evaluations. Unfortunately, the perfect hearing aid does not exist.

However, many hearing aid algorithms can be evaluated with comparison-based quality models. Examples include speech enhancement algorithms that operate on already distorted input signals, like noisy and/or reverberant speech. In these evaluations, the original, clean speech recording can serve as a reference signal, and comparison signals are generated by adding noise or reverberation. Such a comparison-based method may also be applicable in the case of frequency-lowering algorithms. Frequency-lowering will always degrade the naturalness of the input sounds. If subjective sound quality ratings are dominated by the perceived naturalness and not influenced by the possibly improved speech intelligibility or other positive effects of the frequency compression, comparison-based quality models using the unprocessed input signal as a reference appear potentially qualified as predictors of sound quality also for this class of hearing aid algorithms.

### Objective evaluation of frequency-compressed speech

NFC is a viable choice as an amplification solution for hearing impaired listeners with severe to profound high-frequency hearing loss. Since sound quality is one of the most important factors determining the overall satisfaction and acceptance of hearing aid users [21]–[24], the negative impact on sound quality by NFC must remain small enough to be overshadowed by the positive effects such as improved intelligibility in order to achieve acceptance and overall preference over conventional processing. Objective sound quality models are useful in this context as they can assist in the initial specification of NFC parameters that achieve a balance between intelligibility enhancement and quality degradation. In the present study, a number of established as well as rather new, mostly perceptual speech and audio quality models, including yet unpublished extended versions, have been applied to predict subjective sound quality ratings of nonlinear frequency-compressed speech [11]. It should be noted that none of the applied models was designed for the present application and some of them were particularly optimized for different experimental conditions, like (monaural) headphone presentation instead of loudspeaker presentation as used in the present study. In spite of this caveat, promising predictor candidates will be identified below.

## Subjective Speech Quality Measurement

Speech signals and subjective quality ratings used to test the quality models were obtained from an earlier study [11] and shall be described only briefly here. See [11] for a more detailed description.

**Figure 2:**
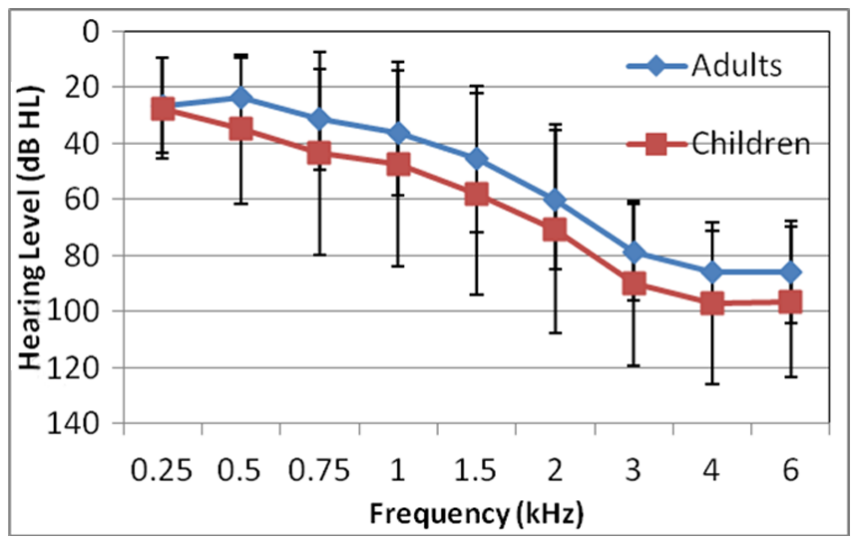
Mean and standard deviations of the pure-tone air conduction thresholds for hearing impaired adults and children.

Speech quality ratings were obtained from a group of 12 normal hearing adults (ages 21–27 years, mean = 24 years), 12 normal hearing children (ages 8–18 years, mean = 12 years), 12 hearing impaired adults (ages 50–81 years, mean = 69 years), and 9 hearing impaired children (ages 8–17 years, mean = 12 years). The listeners with hearing impairment also participated in a field study with NFC hearing aids [8]. All normal hearing listeners had pure tone thresholds of 20 dB HL or better across the audiometric frequency range. Figure 2 depicts the mean and standard deviation of the pure tone thresholds for hearing impaired adults and children. While the children had higher absolute thresholds than adults, statistical analysis revealed there was no significant interaction between frequency-specific thresholds and age [8], [11].

A database of frequency-compressed speech samples was created to obtain the subjective quality ratings. The database was constructed by first programming a prototype NFC hearing aid with specific cutoff frequency (*F_c_*) and compression ratio (CR) parameters, placing this hearing aid in a Brüel and Kjær portable anechoic test box, and recording the output of the hearing aid separately in response to two male speech samples and two female speech samples. A total of five different NFC settings were investigated: (1) *F_c_* = 4000 Hz, CR = 2∶1; (2) *F_c_* = 3000 Hz, CR = 2∶1; (3) *F_c_* = 3000 Hz, CR = 6∶1; (4) *F_c_* = 3000 Hz, CR = 10∶1; and (5) *F_c_* = 2000 Hz, CR = 2∶1.

Hearing aid recordings were later played back to the bilaterally aided study participants in a sound booth through a loudspeaker positioned at 0° azimuth and at a distance of 1.5 m. The playback level was initially presented at 65 dB SPL and then individually adjusted, if needed, to the subject’s most comfortable level (MCL) [11]. Calibration was performed using the Brüel & Kjær Sound Level Meter (SLM), Type 2270. Speech quality ratings were obtained using the ITU standard MUltiple Stimulus with Hidden Reference and Anchors (MUSHRA, [25]) protocol mediated by custom software. The MUSHRA protocol uses a numerical quality rating scale from 0 to 100 together with five verbal categories from “bad” to “excellent” as anchors for orientation. In this method, a “reference stimulus” was presented, which was the unprocessed male or female speech sample, while eight “test stimuli” were randomly associated with the five NFC-processed stimuli, the original signal itself (“hidden reference”), and a low-pass version and a peak-clipped version of the original signal, respectively. The low-pass version was obtained by passing the original signal through a 10^th^ order Butterworth filter with a cutoff frequency of 2000 Hz, while the peak-clipped version was obtained through hard-clipping the original stimulus at 25% of its peak value. These stimuli served as “anchor” stimuli that represent sound with poor quality, as recommended by [25]. In the MUSHRA method, all stimuli are rated simultaneously using graphical slider buttons. Participants were instructed to listen to each of these stimuli in comparison to the reference, by clicking on the corresponding icons of the software’s GUI and adjust the rating sliders such that a satisfactory quality rating of all eight stimuli was achieved. Participants were allowed to re-listen to any stimulus and re-adjust their quality ratings until they were satisfied with the final set of ratings.

Parsa *et al.* [11] have conducted a detailed statistical analysis of the subjective data collected through this procedure. A high degree of intra- and inter-rater reliability was observed with the speech quality ratings across both normal and hearing impaired groups. As such, this database was used to benchmark the performance of different objective speech quality estimators in predicting the quality of frequency-compressed speech.

## Objective Speech Quality Measurement

This paper evaluated twenty-nine objective quality measures including sub-variants in order to benchmark model performance for prediction of sound quality with NFC processing. Table 1 provides a summary of the models/measures tested, and technical information on the nature of each model is provided below.

**Table 1:** Quality models and measures used in this study.

Model/measure name	acronym	authors	ref.	measure(s)	HI?	no.
Hansen model		M. Hansen & Kollmeier	[37]	qc+W+B	−	M1
				qc+W-B	−	M2
				qc-W+B	−	M3
				qc-W-B	−	M4
Hansen model extended for hearing impaired		Huber	unpub.	qc+W+B (HI)	✓	M5
				qc+W-B (HI)	✓	M6
				qc-W+B (HI)	✓	M7
				qc-W-B (HI)	✓	M8
PEMO-Q	PEMO-Q	Huber & Kollmeier	[39]	PSM_fb+B	−	M9
				PSM_fb-B	−	M10
				PSM_lp+B	−	M11
				PSM_lp-B	−	M12
PEMO-Q extended for hearing impaired	PEMO-Q-HI	Huber	unpub.	PSM_fb+B (HI)	✓	M13
				PSM_fb-B (HI)	✓	M14
				PSM_lp+B (HI)	✓	M15
				PSM_lp-B (HI)	✓	M16
Hearing Aid Speech Quality Index	HASQI	Kates & Arehart	[19]	HASQI_lin_	✓	M17
				HASQI_nonlin_	✓	M18
				HASQI_comb_	✓	M19
Perceptual Evaluation of Speech Quality (ITU-T Rec. P.862-2)	PESQ	ITU-T/Beerends et al.	[14], [31]	PESQ-LQO	−	M20
				PESQ-LQO (WB)	−	M21
Loudness Pattern Distortion	LPD	Guo & Parsa	[36]	LPD	−	M22
Moore quality model		Moore & Tan	[33]–[35]	*D*	−	M23
				*R_nonlin_*	−	M24
				*S_overall_*	−	M25
Itakura-Saito Distance	ISD	Itakura & Saito	[26]	ISD	−	M26
Log-Area Ratio	LAR	Quackenbush et al.	[28]	LAR	−	M27
Log-Likelihood Ratio	LLR	Itakura	[27]	LLR	−	M28
Weighted Spectral Slope Distance	WSSD	Klatt	[30]	WSSD	−	M29

(“ref”.: reference; “HI?”: Does model account for hearing impairment? Yes: ✓; no: −) Quality measure suffixes: fb/lp: modulation filterbank/lowpass model version; +/−W: with/without frequency band weighting; +/−B: with/without asymmetric weighting of differences (“Beerends weighting”).

The objective models investigated in this paper are considered to be “double-ended” or “intrusive” in their operation. Double-ended models compare features extracted from the signal under test to those extracted from an undistorted reference version of the same signal. The comparisons quantify the degree of perceptual overall difference or similarity. In contrast, single-ended models do not require a reference signal. The models in Table 1 can be broadly grouped into: (1) metrics based on speech production model parameters, and (2) metrics based on the comparison of “internal representations” obtained with computational models of auditory processing. Double-ended models were chosen for this work, because the subjective quality ratings were obtained using reference signals as well, according to the MUSHRA protocol.

Examples of objective models in the speech production model parameters include the Itakura-Saito Distance (ISD) [26] and the Log-Likelihood Ratio (LLR) [27], which calculate the weighted similarity between the linear prediction coefficients extracted from the distorted and reference speech signals, and the Log-Area Ratio (LAR) [28] measure, which computes the distance between the area ratio coefficients. Software implementations of ISD, LAR, and LLR were taken from a toolbox provided by Hansen and Pellom [29].

A basic objective measure that uses an auditory model is the Weighted Spectral Slope Distance (WSSD) [30] measure. It uses a psychoacoustically motivated bank of critical-band filters to decompose the speech signals, and weights the differences between the slopes of the log magnitudes in each band to produce the final quality measure. Again, the software implementation of the WSSD measure was taken from the Hansen and Pellom toolbox.

The method for the Perceptual Evaluation of Speech Quality (PESQ; ITU-T Recommendation P.862 [14]) and its wide band extension (PESQ-WB; ITU-T P.862.2 [31]) are perhaps the most popularly used objective quality assessment methods incorporating an auditory model. Conceptually, the PESQ technique computes the frame-by-frame internal representations of test and reference signals by first computing the power spectra, and then applying frequency and intensity warping functions based on Zwicker’s loudness model [14].

Moore & Glasberg have developed a different loudness model [32], characterized by the computation of excitation patterns at the output of each auditory filter and the subsequent computation of the specific loudness pattern. Moore *et al.* [33]–[35] applied the Moore & Glasberg model and derived an objective quality metric based on comparisons of the excitation patterns of original and distorted signals. Two intermediate measures of linear (spectral) and nonlinear distortions (*D* and *R_nonlin_*, respectively) are computed separately and combined to an overall subjective score predictor *S_overall_* in the end. For the Moore quality model, a custom software implementation was developed. Correlations between model output measures and mean subjective quality ratings will be reported for *D*, *R_nonlin_* and *S_overall_*.

Chen *et al.* [36] devised an objective measure based on the Moore & Glasberg loudness pattern differences between the reference and degraded speech samples. These differences were used to estimate sound quality in the degraded sample versus the reference sample. This measure was computed using custom software.

The speech quality model of Hansen & Kollmeier [37] (referred to as the “Hansen model” below) employs the auditory processing model of Dau *et al.* [38], “PEMO” (for “PErception MOdel”), for the computation of internal representations of test and reference signals. The bandwidth of the PEMO peripheral (gammatone) filterbank was adapted to telephone-bandpass-filtered speech. A “band importance weighting” function is applied to the frequency channels of the internal representations to emphasize higher frequency channels. The overall correlation between the weighted internal representations determines the quality measure qc.

The audio quality model PEMO-Q of Huber and Kollmeier [39] extends the Hansen model to include quality assessment of general, wideband audio signals (including speech) with low to very high audio qualities. By default, it uses the more recent version of the Dau model [40], with the option of selecting the earlier (much faster) version. The two model versions differ with respect to the processing of amplitude modulations: In [38], the final step of auditory preprocessing (i.e. before the decision stage) is an 8 Hz-lowpass filter, whereas in [40], this lowpass filter is replaced by a filter bank. The band importance weighting function used in the Hansen model was not adopted for PEMO-Q.

PEMO-Q outputs two quality measures: The Perceptual Similarity Measures PSM and PSM_t_. The second variant evaluates the temporal course of the instantaneous audio quality, derived from a frame-wise correlation of internal representations, nonlinearly mapped to the overall quality estimator PSM_t_. In contrast, the PSM is the overall correlation of the complete internal representations. The PSM_t_ is more sensitive to small distortions and more independent of the type of input audio signal [39]. On the other hand, the “simpler” PSM seems to be more “robust” and more generally applicable, especially for low-to intermediate audio quality. For example, speech enhancement algorithms have been evaluated with this measure and showed good correlations with subjective quality ratings [41]–[43]. Since speech samples with rather strong quality differences are considered in this study, only the PSM was included here.

By default, PESQ and PEMO-Q apply an asymmetric weighting of differences between internal representations, putting more weight on “added” or increased elements of the internal representation of the distorted signal than on “missing” or attenuated ones [44]. This step is handled as an option for the Hansen model and PEMO-Q and has been switched on and off in this study. The second binary setting option of PEMO-Q that has been varied is the manner of amplitude processing in the auditory processing model (modulation lowpass vs. filterbank). This leads to four PEMO-Q output measure variants that have been tested in total. Since a benefit of the band importance function used in the Hansen model could only be found in the prediction of speech quality in the context of telecommunication, the Hansen model was also tested with the weighting function deactivated in this study. Hence, four versions of the Hansen model have been tested in total: with and without asymmetry weighting of internal representations, and with and without applying the band importance weighting function.

Finally, the Hearing Aid Speech Quality Index (HASQI) by Kates and Arehart [19] was employed. In the computation of the HASQI, a relatively simple auditory processing model is applied to provide internal representations in terms of neural firing rates. Effects of possible sensorineural hearing losses are modeled by estimating the contributions of inner hair cell (IHC) and outer hair cell (OHC) losses to a total hearing loss given by the audiogram. Auditory filter bandwidths are increased and compression ratios are reduced depending on the OHC loss estimate. The effect of IHC loss is modeled by linear signal attenuation in each auditory filter before compression. From the comparison of internal representations of test and reference signals, two intermediate quality metrics are computed, quantifying the amount of linear and non-linear distortions, respectively. The final overall quality index is the product of the linear and the nonlinear distortion metrics. Again, correlations with subjective quality ratings will be reported for linear and nonlinear metrics and overall quality index, referred to as HASQI_lin_, HASQI_nonlin_ and HASQI_comb_, respectively.

In summary, the models and measures for speech and audio quality listed in Table 1 have been applied to the signals of the database.

The versions of PEMO-Q and the Hansen model with extensions for hearing impaired data have not been published before and will therefore be discussed in the next section.

### Extended versions of PEMO-Q and the Hansen model for hearing impaired

**Figure 3:**
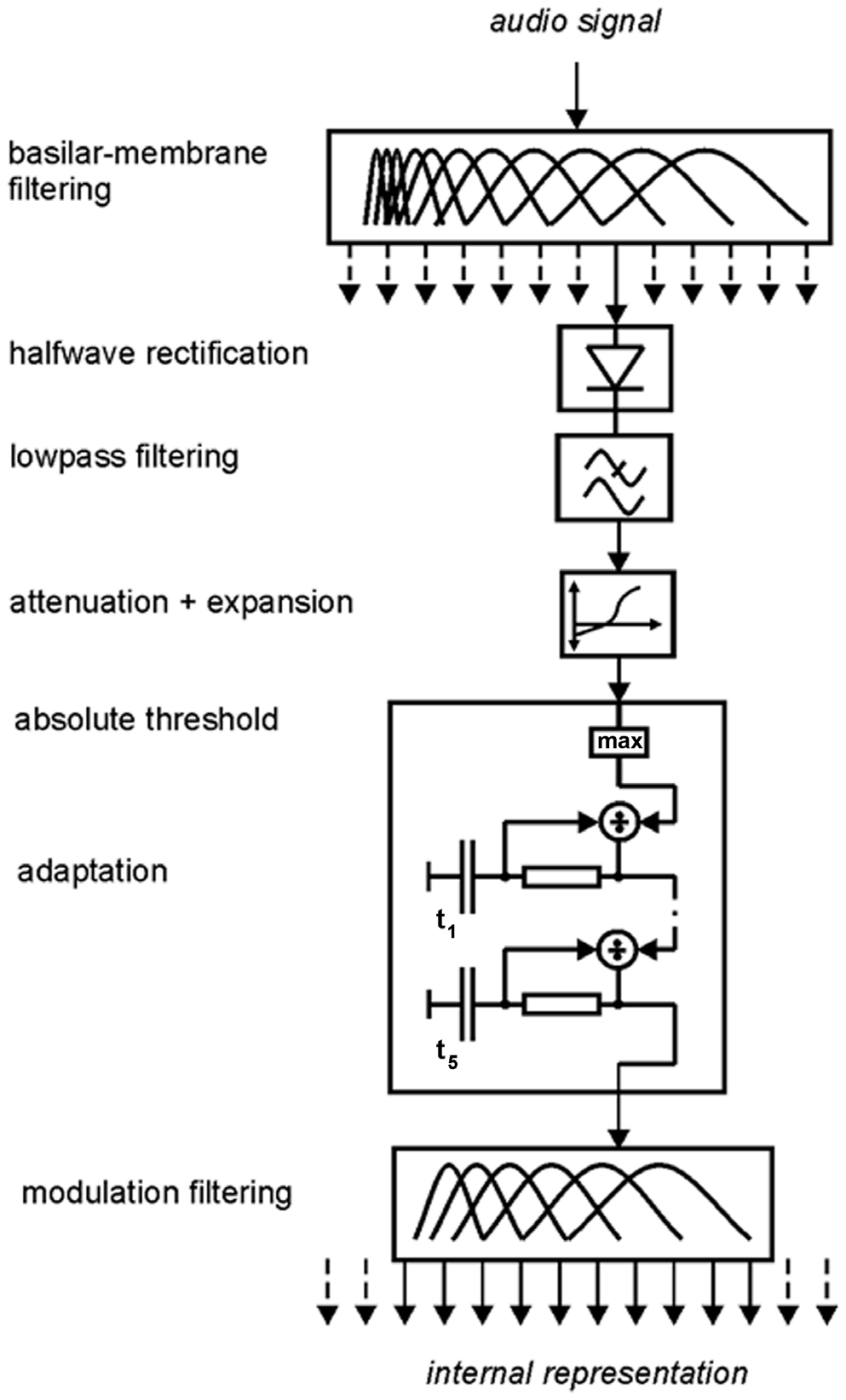
Schematic plot of the auditory processing model PEMO, modified to simulate normal and impaired hearing (after [40] and [45]).



Modifications of the underlying auditory model of the audio quality prediction methods PEMO-Q and its predecessor, the speech quality model of Hansen and Kollmeier, for describing sensorineural hearing losses have been suggested by Derleth *et al.* [45]. One of their suggestions was to insert an instantaneous expansion and attenuation stage before the adaptation and compression stage in the PEMO (Figure 3), to account for a loss of sensitivity and reduced dynamic compression. This suggestion has been adopted and implemented in the extended version of PEMO-Q and the Hansen model. Given an audiogram as the input, the hearing thresholds in dB HL at audiometric frequencies are interpolated internally to the center frequencies of the model’s peripheral filterbank. The inserted processing stage operates on the output of each of these filters, after envelope extraction. The total hearing loss in the respective channel is decomposed into contributions attributed to losses of inner (IHCL) and outer hair cells (OHCL), respectively, as shown in the equation below [32]:

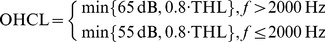
(2)

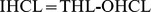



**Figure 4:**
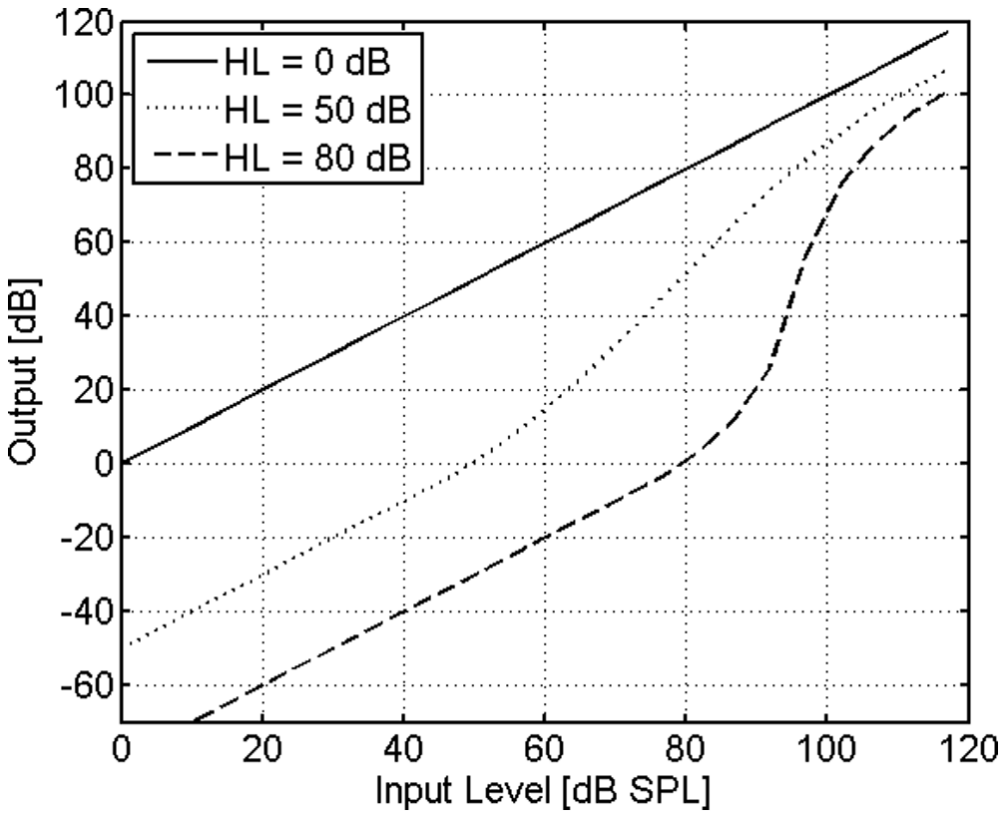
Input/output functions of the expansion and attenuation model stage for normal hearing (solid line) and impaired hearing with an assumed total hearing loss of 50 dB (dotted line) and 80 dB (dashed line).

In the model stage, the signal amplitude is attenuated by the amount of IHCL. Then, it is raised to the power *k*, which itself is a function of the input signal amplitude *I* and OHCL. The attenuation and expansion operations are performed instantaneously (i.e. sample-by-sample). For simulating normal hearing, *k* equals 1 for any *I*. For simulating impaired hearing, *k*(*I*) becomes larger than 1 and increases with OHCL. The input/output function of this stage is shown in Figure 4 for normal hearing and two hearing losses. The parameters of the input/function were chosen such that its shape matches the inverse function of the assumed input/output function of the unimpaired cochlea proposed by Moore [46]. Again, model parameters were not adjusted in the present study.

## Results

The performance of the objective measures was characterized by their correlations with averaged subjective quality ratings of all processing conditions (including hidden reference and anchors), quantified by Pearson’s linear correlation coefficient *r* and Spearman’s rank correlation coefficient *rs*. Quality measure values were taken directly as output by the models and not transformed by, e.g., nonlinear regression functions.

As there were no systematic differences between the quality ratings of normal hearing adults and children, quality ratings were averaged across the complete group of normal hearing subjects. Consequently, results are stated for three groups of subjects separately: (1) normal hearing (adults and children), (2) hearing impaired children, and (3) hearing impaired adults. In addition to averaging across subjects, quality ratings were also averaged across different talkers per processing condition. Results will be stated for both levels of averaging. For clarity, results per subject group will be presented separately in succession and only for a selection of measures. The complete results are reported in the Appendix S1. The relation between subjective ratings and corresponding model predictions will be illustrated by scatter plots for the best performing quality measures.

### Results for normal hearing

Table 2 shows linear and rank correlations between selected model predictions and mean subjective ratings obtained from normal hearing subjects. (The complete correlation table is given in the Appendix S1) In this table, the suffixes of the PSM and qc measures have the following meaning: fb/lp indicate the modulation filterbank/lowpass version of PEMO-Q respectively, +/−W indicate the activation/deactivation of the frequency-band weighting, and +/−B denote activation/deactivation of the asymmetric weighting of internal representation differences (“Beerends weighting”). When frequency weighting is deactivated in the Hansen model, and the modulation lowpass version is used in PEMO-Q, these models become very similar. They mainly differ in the bandwidth of the peripheral filterbank. It must be noted that because the results obtained with the –B option tended to be better than with option +B, the latter are only reported in the complete set of results given in the Appendix S1.

**Table 2:** Results of the quality predictions (including anchor conditions) for the normal hearing subjects, expressed by linear correlation coefficients and rank correlation coefficients (*italic*).

measure	ind.	av.
qc+W-B (M2)	0.84	0.86
	*0.89*	*0.93*
qc-W-B (M4)	0.84	0.87
	*0.84*	*0.95*
PSM_lp-B (M12)	0.85	0.88
	*0.84*	*0.91*
PSM_fb-B (M10)	0.88	0.89
	*0.92*	*0.98*
HASQ_comb_ (M19)	*0.54*	*0.54*
	*0.24*	*0.19*
PESQ (M21)	0.64	0.64
	*0.41*	*0.45*
*S_overal_* (M25)*_l_*	0.76	0.76
	*0.63*	*0.64*
LPD (M22)	−0.75	−0.77
	−*0.64*	−*0.62*
LAR (M27)	−0.83	−0.84
	−*0.74*	−*0.71*

Subjective ratings were averaged across subjects. Middle column: correlations based on ratings for individual talkers (ind.); right column: ratings averaged across talkers (av.). Quality measure suffixes: fb/lp: modulation filterbank/lowpass model version; +/−W: with/without frequency band weighting; +/−B: with/without asymmetric weighting of differences (“Beerends weighting”).

It can be seen from Table 2 that the correlation coefficients are reasonable for several of the objective quality metrics, especially when averaged across different talkers per processing condition (right column “av.”) Four of the five measures with highest correlations are PEMO-Q and related measures (PSM…, qc…), complemented by the LAR (M27 of Table 1) measure. The latter, however, shows only a rather poor rank correlation with the subjective ratings. Good correlations, especially rank correlations, are achieved when ratings are averaged across different talkers per processing condition because averaging reduces the statistical noise of the ratings and thus increases the reliability of the data. On the other hand, the significance of the correlation coefficients gets lower as the number of data points is reduced to only eight.

**Figure 5:**
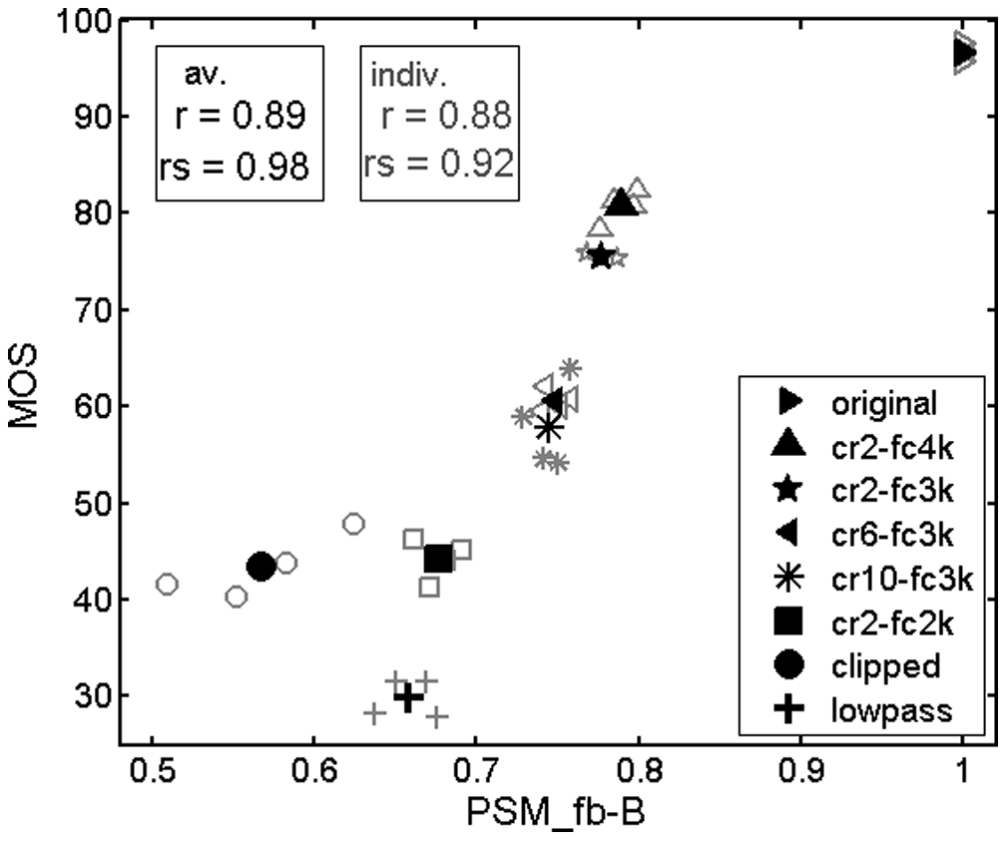
Scatter plot of the results obtained with the PEMO-Q measure PSM_fb-B for the normal hearing subjects. Subjective ratings (Mean Opinion Scores – MOS) are plotted versus corresponding model predictions. r: linear correlation, rs: rank correlation.

Figure 5 shows a scatter plot of the results obtained with the measure PSM_fb-B (M10). It must be noted that the results for two of the four talkers processed by the NFC condition cr2_fc3k were not available due to a labelling error and a processing error of the concerned test files. It can be observed from this figure that the quality ratings for the NFC test conditions are bordered by the ratings of the high quality (hidden) reference and the low quality anchor stimuli (lowpass-filtered and clipped speech, respectively). The scatter plot reveals that the model underestimates the perceived quality of the clipped speech samples whose predicted quality was lower than that of the lowpass-filtered samples, whereas the subjects ranked the qualities of these conditions in an opposite manner. Another noteworthy difference between model predictions and the subjective ratings is the larger predicted quality difference between the reference condition and the processed conditions compared to the differences among processed conditions.

Both subjects and model rate the frequency-compressed signals with the lowest cutoff frequency 2 kHz (condition cr2-fc2k) very poorly compared to the other frequency compression conditions. A possible cause for this non-linear relation between compression cutoff frequency and perceptual effect could be the alteration of speech formant frequencies and/or the interference of down-shifted higher frequency contents with these formants. Vowel formants have significant energy in the second and third formants in approximately the 1000–4000 Hz region for female speech and approximately 750–3000 Hz for male speech, depending on the specific vowel and talker. Recalling that both male and female speech samples were used, it is reasonable to conclude that the cr2-fc2k setting was strong enough to lower significantly the upper formants of many vowels for both male and female speech and was therefore much more noticeable than settings that likely did not disrupt the harmonic relationships within vowels, such as those that used a 4000 Hz cutoff frequency.

Figure 5 shows that in general, the influence of the talker on the measured and predicted quality ratings is very small, except for the clipping condition. Here, the influence of the talker is overestimated by the PSM measure.

### Results for hearing impaired children

**Figure 6:**
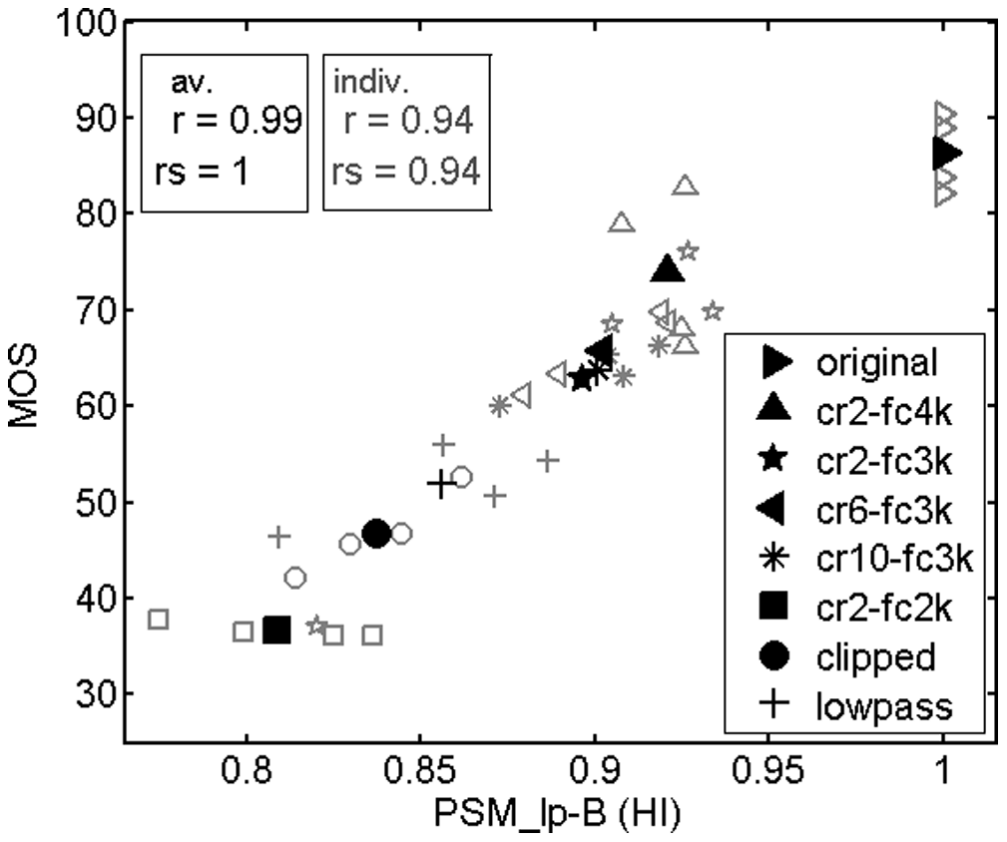
Same as **Figure 5**, but for hearing impaired children.

The results obtained for the hearing impaired children are shown in Table 3 and Figure 6. According to the correlations with subjective ratings (Table 3), the prediction accuracy of the models for hearing impaired listeners for separate talkers was very good (*r* up to 0.94), and even higher when averaged across talkers (*r* up to 0.99). The slightly better performance of the modulation filterbank version of PEMO-Q in the prediction of normal hearing subjects’ ratings (Table 2) was not confirmed by the results obtained with hearing impaired children (Table 3).

**Table 3:** Quality prediction results (linear and rank correlation (*italic*) coefficients) for the hearing impaired children group.

measure	NH models		HI models	
	ind.	av.	ind.	av.
qc+W-B (M2, M6)	0.58	0.59	0.87	0.90
	*0.78*	*0.88*	*0.89*	*0.93*
qc-W-B (M4, M8)	0.82	0.87	0.90	0.95
	*0.80*	*0.91*	*0.91*	*0.93*
PSM_lp-B (M12, M16)	0.87	0.91	0.94	0.99
	*0.86*	*0.95*	*0.94*	*1.00*
PSM_fb-B (M10, M14)	0.84	0.87	0.94	0.97
	*0.87*	*0.93*	*0.95*	*0.95*
HASQ_comb_ (M19)	*0.73*	*0.76*	*0.81*	*0.85*
	*0.80*	*0.69*	*0.87*	*0.88*
PESQ (M20)	0.90	0.92		
	*0.90*	*0.83*		
*S_overall_* (M25)	0.83	0.85		
	*0.90*	*0.88*		
LPD (M22)	−0.51	−0.55		
	−*0.53*	−*0.57*		
LAR (M27)	−0.61	−0.63		
	−*0.65*	−*0.67*		

Results for the PEMO-Q measure PSM and the related measure qc are given for normal hearing (NH) and hearing impaired (HI) model versions. Quality measure suffixes: fb/lp: modulation filterbank/lowpass model version; +/−W: with/without frequency band weighting; +/−B: with/without asymmetric weighting of differences (“Beerends weighting”).

Figure 6 depicts a scatter plot for PSM_lp-B, hearing impaired (HI) version (M16) and subjective ratings from hearing impaired children. In contrast to Figure 5, a greater variation of quality ratings and predictions for different talkers can be seen in this figure. Note that the predicted quality of the original, unprocessed condition is always maximal, since this condition is used as the reference by the comparison-based quality models. Hence, possible influences of different talkers cannot be modeled for this condition. Another difference from the normal hearing data concerns the rank order of the three processing conditions with the lowest ratings, i.e. lowpass filtering (LP), clipping (CL) and frequency compression with the lowest cutoff frequency, 2 kHz (cr2-fc2k). Normal hearing subjects ranked LP< CL ≈ cr2-fc2k. The model, in contrast, ranked CL<LP< cr2-fc2k. Hearing impaired children, however, ranked these conditions clearly differently: cr2-fc2k<CL<LP. This rank order was correctly predicted by the quality measure PSM_lp-B (HI) (M16). As a consequence, a very high linear correlation (*r* = 0.99) and a perfect rank correlation (*rs* = 1) between PSM_lp-B and subjective ratings were achieved when averaged across talkers. Finally, the range of PSM_lp-B (HI) (M16) values is smaller compared to PSM_fb-B (M10) values used for normal hearing subjects, while the range of subjective ratings is about the same in both cases. This difference is due to the greater sensitivity of the modulation filterbank version of the auditory model PEMO used for the computation of PSM_fb-B (M10), compared to the modulation lowpass filter version used for computing PSM_lp-B (HI) (M16).

### Results for hearing impaired adults

**Figure 7:**
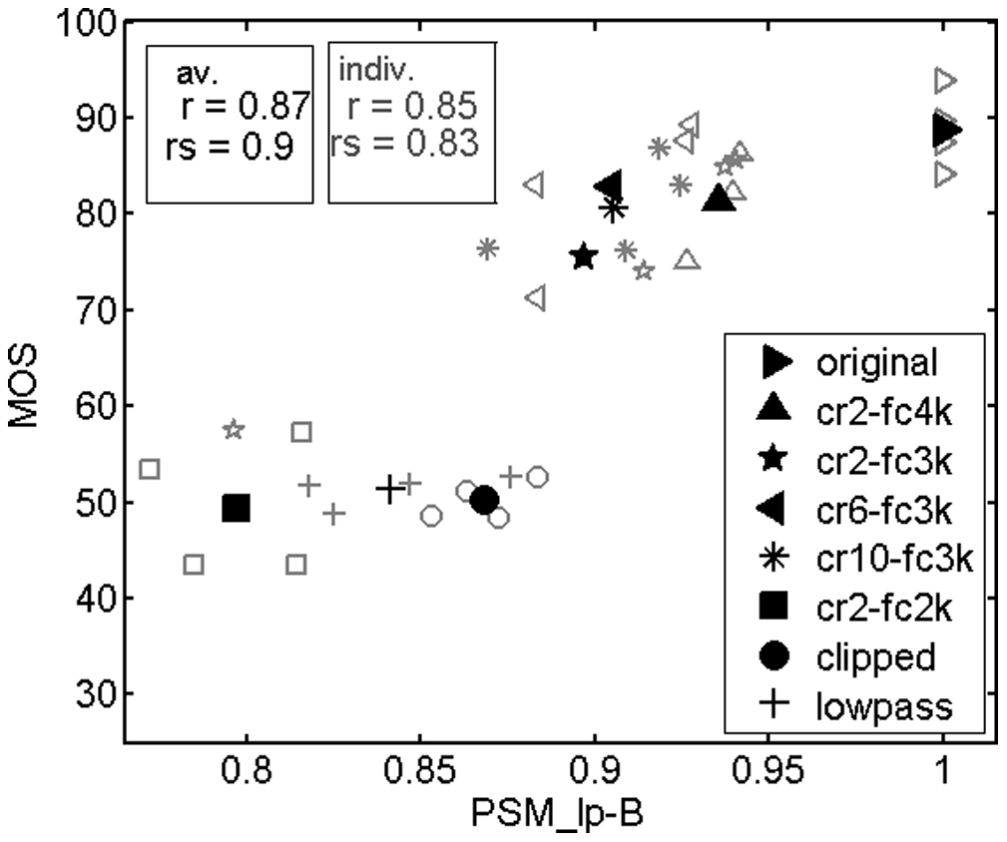
Same as **Figure 5**, but for hearing impaired adults.

**Figure 8:**
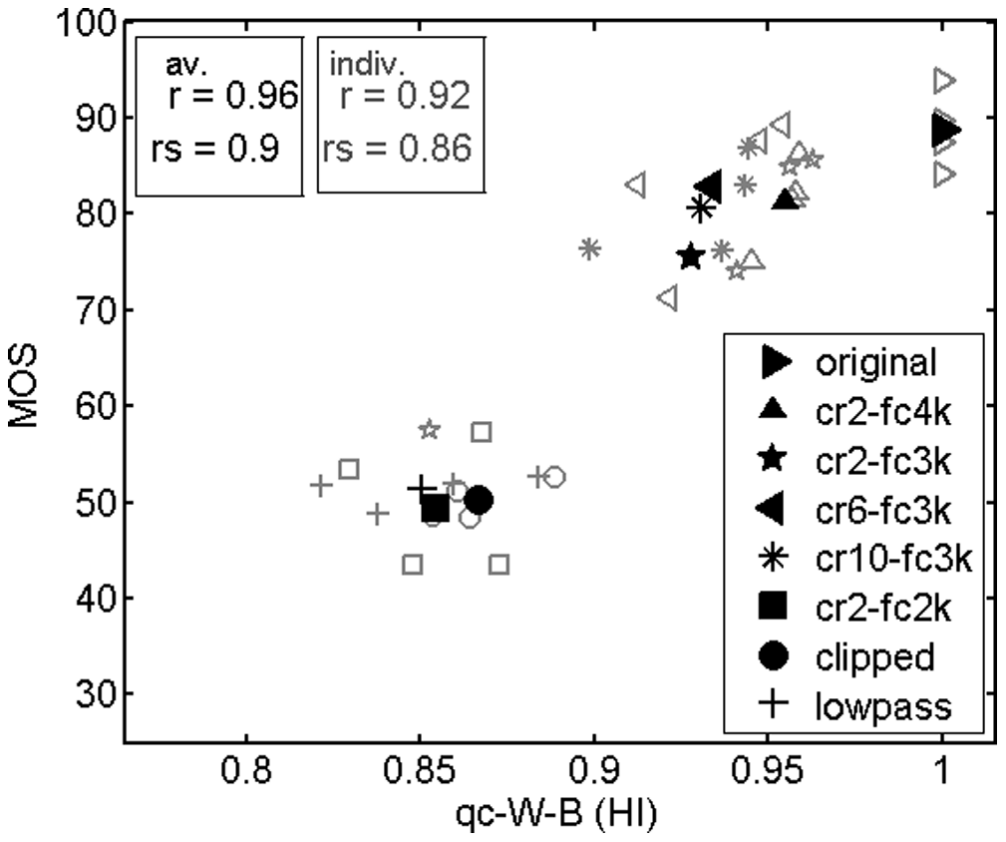
Quality prediction results for the hearing impaired adults group, obtained with quality measure qc-W-B (version for hearing impaired). r: linear correlation, rs: rank correlation.

Hearing impaired adults exhibited a different rating behavior than hearing impaired children on average, although there were no significant differences between the distributions of hearing losses in these two groups (children’s hearing losses were somewhat higher than adults’ hearing losses on average). Adults rated NFC conditions generally higher, except for condition cr2-fc2k, whereas their ratings of the anchor condition (LP, CL) were similar to children’s ratings (compare Figure 7 with Figure 6). As a result, adults’ average ratings of cr2-fc2k, LP and CL conditions were very similar, whereas the quality measure PSM_lp-B (HI) (M16) clearly rated cr2-fc2k<LP< CL, which led to a lower correlation between measured and predicted mean quality ratings (*r* = 0.88) compared to the results obtained for the children (*r* = 0.99). In contrast, a very high correlation was achieved by the related quality measure qc-W-B (HI) (M8) (*r* = 0.96). As described earlier, qc-W-B (HI) (M4, M8) mainly differs from PSM_lp-B (HI) (M12, M16) in the lower cutoff frequency of the peripheral filterbank. Looking at Figure 8, the higher correlation obtained with qc-W-B (HI) (M8) is mainly due to a shift of quality ratings of NFC conditions towards higher values compared to the PSM_lp-B (HI) (M16) values, whereas ratings of the anchor conditions LP and CL basically correspond to the PSM_lp-B (HI) (M16) values. This agrees well with the observed difference in subjective ratings between hearing impaired children and adults. Table 4 summarizes the prediction performances of the different quality measures. Amongst others, it clearly shows the advantage of using models that account for hearing impairment, as evidenced by the higher correlations with subjective quality ratings for most of the evaluations.

**Table 4:** Quality prediction results (linear and rank correlation (*italic*) coefficients) for the hearing impaired adults group.

measure	NH models		HI models	
	ind.	av.	ind.	av.
qc+W-B (M2, M6)	0.65	0.67	0.77	0.79
	*0.68*	*0.74*	*0.75*	*0.74*
qc-W-B (M4, M8)	0.85	0.88	0.91	0.96
	*0.86*	*0.76*	*0.87*	*0.81*
PSM_lp-B (M12, M16)	0.85	0.88	0.86	0.88
	*0.86*	*0.81*	*0.84*	*0.81*
PSM_fb-B (M10, M14)	0.78	0.81	0.82	0.85
	*0.79*	*0.81*	*0.79*	0.86
HASQ_comb_ (M19)	*0.50*	*0.51*	*0.78*	0.79
	*0.53*	*0.62*	*0.86*	0.95
PESQ (M20)	0.62	0.63		
	*0.79*	*0.81*		
*S_overall_* (M25)	0.50	0.51		
	*0.42*	*0.48*		
LPD (M22)	−0.52	−0.57		
	−*0.37*	−*0.43*		
LAR (M27)	−0.62	−0.65		
	−*0.49*	−*0.48*		

Quality measure suffixes: fb/lp: modulation filterbank/lowpass model version; +/−W: with/without frequency band weighting; +/−B: with/without asymmetric weighting of differences (“Beerends weighting”).

## Discussion

The obtained results show that predicting the perceived sound quality of nonlinearly frequency-compressed speech by means of objective, perceptual speech/audio quality models is possible. From the models tested, the PEMO-Q (related) measures, in particular, the Perceptual Similarity Measure (PSM) and the speech quality measure qc of the Hansen model (with deactivated frequency weighting function), showed the highest correlations with mean subjective ratings. These measures represent the most “basic” PEMO-based quality measures with the lowest degree of optimization for a specific task and hence the highest generality and robustness. This is in line with results of other studies where different quality measures have been applied for tasks other than those for which they were originally developed (e.g. [41]–[43]). Through the results of the present study, the generality of abovementioned measures is further proven given that the predicted quality degradations caused by very different kinds of distortions (i.e. frequency compression, amplitude clipping and lowpass filtering) correlated highly with subjective ratings.

A number of objective measures that were previously either applied to speech or audio quality estimation were evaluated in this paper. Of the objective measures based on speech production model parameters (viz. ISD (M26), LAR (M27), LLR (M28)), LAR (M27) performed the best. In the past, these metrics were primarily used for estimating narrowband (0–4 kHz bandwidth) speech quality (e.g. [28], [29]). Since some of the PEMO-based quality measures employed a wider bandwidth, experiments were conducted with implementations of ISD (M26), LAR (M27) and LLR (M28) metrics using an extended 8 kHz bandwidth. Results, however, showed a degradation in the correlation coefficients with subjective ratings for LAR (M27) and LLR (M28) metrics compared to the original versions with 4 kHz bandwidth. Similarly, the LPD (M22) metric was developed and validated for narrowband speech applications [36], and its performance with an extended bandwidth is not investigated in this work and is a worthwhile topic for future study.

Speech quality measures (M22, M25) based on the Moore & Glasberg model ([33]–[36]) performed decently with normal hearing data, but were inferior with the hearing impaired data. A revised model of loudness perception incorporating cochlear hearing loss was proposed by Moore and Glasberg [47], and further investigation would be interesting to determine whether the incorporation of this revised model would lead to an improvement in correlations with hearing impaired subjective data.

The Hearing Aid Speech Quality Index (HASQI, M17–M19) showed good rank correlations and moderate linear correlations with hearing impaired data, but only low correlations with normal hearing data. This is in clear contrast to the results reported in a recent publication on an updated version of the HASQI [48], which was also tested successfully on frequency-compressed speech. The main reason for the lower correlations observed in the present study was found to be a significant quality overestimation of the lowpass condition. The clipping condition was overestimated as well, although to a lesser extent. Without these anchor conditions, linear correlations of the HASQI_comb_ (M19) with subjective ratings increase to 0.83 for normal hearing data (0.85 when ratings are averaged across different talkers per condition), 0.75 (0.78) for hearing impaired adults’ data, and 0.78 (0.82) for hearing impaired children’s data. The HASQI submeasure for linear distortions (M17) achieves very high correlations in this case: 0.93 (0.95), 0.89 (0.95) and 0.95 (0.996) for normal hearing subjects’, hearing impaired adults’ and hearing impaired children’s data, respectively.

In the present application, PESQ (M20, M21) surprisingly achieved poor correlations with subjective ratings from normal hearing listeners, but moderate and even high correlations with ratings from hearing impaired adults and children, respectively. The main reason for the poor results regarding normal hearing listeners was found to be a quality overestimation of the lowpass-filtered items by PESQ. Without this processing condition, the linear correlation obtained with the narrow-band version of PESQ increases to 0.89 (0.92) for normal hearing subjects. Correlations obtained with the wideband version of PESQ are lower in this case.

The successful “basic” PEMO-Q measures required minimal optimization during development, and use a very simple back-end of the underlying quality model. This result is interpreted as a reflection of the validity of the auditory model originally derived by Dau *et al.* [38], [40]. It represents a well-founded model of the “effective” signal processing in the auditory system and has been validated in a wide variety of psychoacoustical experiments [40], [49]–[55]. Its free parameters were adopted from psychoacoustical modeling and kept fixed in the development of the Hansen model and PEMO-Q as well as in the present study. When applied as a front-end for speech and audio quality prediction, only the bandwidth of the peripheral filterbank (i.e. the range of center frequencies) and the modulation filterbank were set appropriately. The bandwidth used in PEMO-Q appears to have been appropriate for use with the NFC data modeled in this study, too, although we did not evaluate impacts of any adjustments to these original settings. In the hearing-impaired adults group, however, better results were obtained with the quality measure qc with its smaller filterbank bandwidth compared to PSM. This suggests that the bandwidth could still be optimized for the present task and possibly lead to a further improvement of the quality prediction accuracy.

As already pointed out before, the higher correlation with the adults’ quality ratings obtained with the measure qc-W-B (HI) (M8) compared to PSM_lp-B (HI) (M16) is mainly due to an upward shift of quality predictions for the 2 kHz-lowpass condition and the frequency-compressed condition with the lowest cutoff frequency of 2 kHz, which is in line with the adults’ rating behavior. The upper cutoff frequency of the peripheral filterbank used in the computation of qc-W-B (HI) (M8) is 4 kHz, whereas it is 15.3 kHz for PSM. The two processing conditions of concern cancel (most of) the energy at frequencies higher than 4 kHz. The measure qc-W-B (HI) (M8) does not “notice” the missing energy above 4 kHz, in contrast to PSM_lp-B (HI) (M16). As a consequence, PSM_lp-B (HI) (M16) detects a larger overall difference between original and processed signals which leads to a lower quality estimate. A possible reason for the better correspondence between hearing impaired adults’ ratings and the qc-W-B (HI) (M8) predictions could be a reduced sensitivity of these subjects for missing energy at frequencies above 4 kHz, or/and a lower perceptual weighting of this degradation component when rating the overall quality. Apparently, the hearing impaired children of this study were more sensitive or less tolerant towards the quality degrading effects of frequency compression than the hearing impaired adults. They rated the quality of frequency-compressed speech samples clearly lower than the adults did, although the distributions of hearing losses as described by audiograms are similar in these two groups; the children’s hearing losses are in fact somewhat higher on average than the adults’ hearing losses. There are two factors that may be related to this difference between age groups. First, age-related auditory and/or cognitive declines not revealed by the audiogram, but influencing quality perception might be factors related to this result. Moreover, age-dependent different experiences with and expectations on the quality of sound reproducing systems might play a role and have been speculated before [56]. However, to the knowledge of the authors, there is no data yet that would support such hypotheses, so these considerations remain speculative. Second, as is clinically typical, we provided the children with a higher level of gain and output in their hearing aids than was provided to adults [57]. For this reason, the children in this study may have had enhanced access to low-level effects in the processed signals and may have responded accordingly in their perceptual ratings. Again, this is a speculation and further study would be required to clarify whether this is a factor.

The fact that similar quality rating scores were obtained from normal hearing adults and children does not contradict the hypothesis of an age-related effect, because normal hearing adults in this study were much younger (mean = 24 years) than hearing impaired adults (mean = 69 years). However, in the present data, not all kinds of distortions were rated differently by older listeners, but mainly the frequency-compressed items. Lowpass-filtered and clipped samples received similar quality ratings from children and adults. The same is observed with the quality measure qc-W-B (HI) (M8), in contrast to PSM_lp-B (HI) (M16) for reasons explained above. As a result, qc-W-B (HI) (M8) correlates better with hearing impaired adults’ quality rating scores, whereas PSM_lp-B (HI) (M16) is better suited to predict the hearing impaired children’s ratings. However, the results obtained from hearing impaired adults and corresponding quality predictions obtained with the measure qc-W-B (HI) (M8) might represent a rather special data subset. In contrast, the measure PSM_lp-B (HI) (M16) appears to have a higher degree of generality and is thus recommended for general application.

### Effect of PEMO-Q options

#### Modulation processing

A clear, consistent effect of the kind of modulation processing stage in PEMO-Q on the prediction accuracy could not be found in the results of the present study. Correlations achieved with the modulation lowpass and modulation filterbank versions of the PEMO-Q quality measure PSM, i.e. PSM_lp and PSM_fb, respectively, were mostly comparable and not consistently ranked with regard to subject group, individual vs. averaged talkers, and linear vs. rank correlation coefficients. Since the computational effort required for PSM_fb is about ten times higher than for PSM_lp, the latter might be preferred for practical applications.

#### Asymmetric weighting of internal representation differences

**Figure 9:**
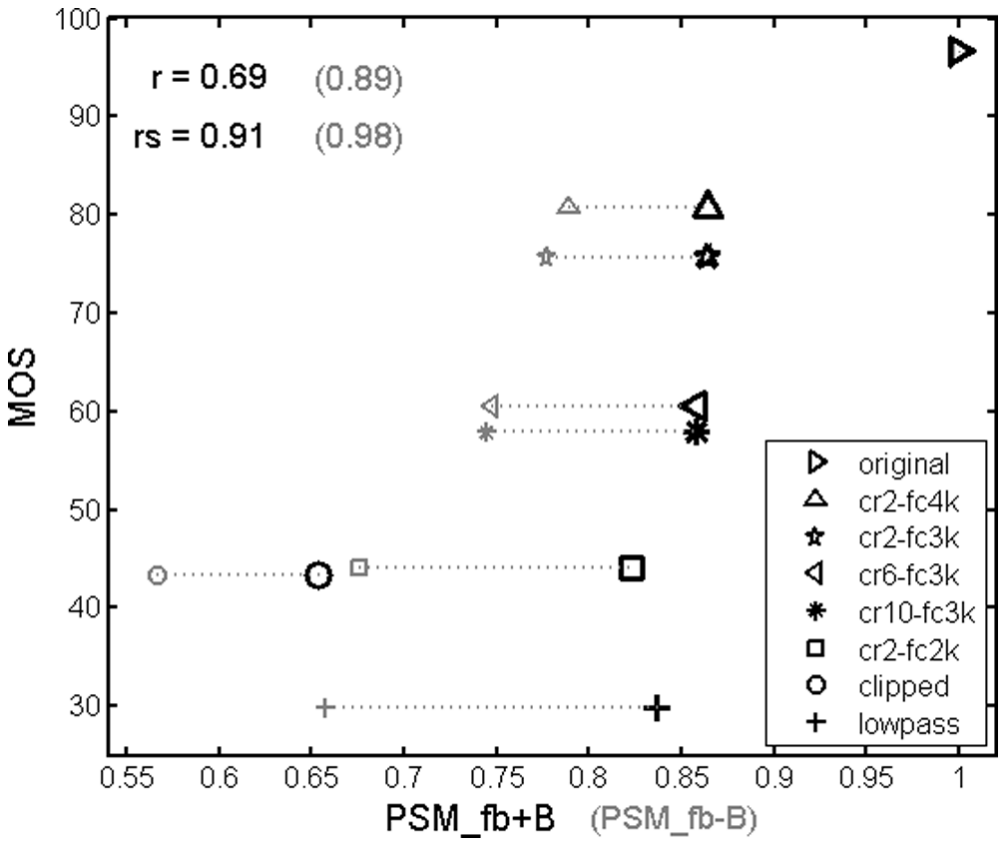
Effect of PEMO-Q model option +B (partial compensation of differences between test and reference internal representations) on quality estimates for the normal hearing subject group. Gray, small symbols: Results obtained with option –B (i.e. without partial compensation); black, large symbols: results obtained with option +B.

The first application for which PEMO-Q was originally developed and optimized was the evaluation of audio codecs. For that purpose, a partial, asymmetric assimilation of internal representations of test and reference signals, equivalent to Beerends’ asymmetry weighting of internal representation differences [44], was introduced into the quality model (option +B). In this procedure, negative deviations of the internal representation of the test signal from the reference are partially compensated (differences are halved), whereas positive deviations remain unchanged. This approach follows the hypothesis that “missing” components in a distorted signal are perceptually less disturbing than “additional” ones. This processing step was found to improve the quality prediction accuracy of PEMO-Q for audio codecs [58]. In the present application, however, better results were obtained without this processing step. The reason for this result is that the negative effect of strong frequency compression and lowpass filtering on sound quality by cancelling energy at medium to high frequencies (>3 kHz) is over-compensated by the assimilation procedure. Consequently, quality estimates are shifted towards higher values disproportionately with increasing frequency compression (i.e., increasing compression ratio and decreasing cutoff frequency) and for the 2 kHz-lowpass filter condition. Figure 9 illustrates this effect with the example of quality prediction results obtained with the quality measure PSM_fb (M9, M10) for the normal hearing subject group with and without assimilation of internal representations (+/−B).

## Summary and Conclusions

In this paper, we presented results from a study on quality prediction of frequency-compressed speech in hearing aids by different objective speech and audio quality measures. Objective measures based on the audio quality model PEMO-Q and the related speech quality measure qc of the Hansen model achieved good correlations with averaged subjective ratings. The extension of these models incorporated hearing impairment, and improved prediction accuracy for quality ratings from hearing impaired subjects. Hearing impaired adults and children rated the quality of frequency-compressed speech differently. Best prediction results were achieved with different quality measures per subject group; adults’ ratings were predicted better with a model that analyzes a smaller frequency bandwidth. Optimizations of bandwidth and possibly further model parameters were not carried out in the present study, but are considered to bear significant potential for further improvements. The HASQI model was also evaluated, and revealed consistently high results across subject groups for the linear submeasure of this index. Again, further evaluation could reveal optimization to the main HASQI model to further improve its performance.

In summary, the present study reports first-of-a-kind results on a broad range of objective sound quality evaluation models of frequency-compressed speech. Further studies are needed to validate the performance of the PEMO-Q quality measures for this application with a larger group of hearing impaired listeners and across more NFC parameter settings. Moreover, the predictability of perceived quality of other sound stimuli than speech, such as music, should be investigated. In addition, the objective modeling of sound quality for frequency lowering schemes other than the NFC scheme evaluated here would require further investigation. In addition, possible influences of age and acclimatization to frequency-compressed sound will have to be investigated and modeled. Research on this topic is driven by the fact that a successful objective speech quality model will have the potential to serve as a valuable supplemental tool for the fitting and evaluation of frequency lowering algorithms in hearing instruments.

## Supporting Information

Appendix S1 (DOCX)
