# Supplementary material for: Predicting the Perceived Sound Quality of Frequency-Compressed Speech
Source: PLoS One. 2014 Nov 17;9(11):e110260. doi: 10.1371/journal.pone.0110260 (PMC4234248; doi:10.1371/journal.pone.0110260)
Supplement: Appendix S1 — (DOCX) [file pone.0110260.s001.docx]

# Appendix

**Complete model correlations**

| **measure** | **NH subjects** | | **HI adults** | | | | **HI children** | | | |
| --- | --- | --- | --- | --- | --- | --- | --- | --- | --- | --- |
|  | **NH models** | | **NH models** | | **HI models** | | **NH models** | | **HI models** | |
|  | **ind.** | **av.** | **ind.** | **av.** | **ind.** | **av.** | **ind.** | **av.** | **ind.** | **av.** |
| qc+W+B (M1, M5) | 0.60 | 0.65 | 0.46 | 0.49 | 0.67 | 0.69 | 0.61 | 0.67 | 0.87 | 0.93 |
|  | *0.40* | *0.41* | *0.41* | *0.33* | *0.71* | *0.74* | *0.47* | *0.55* | *0.89* | *0.93* |
| qc+W-B (M2, M6) | 0.84 | 0.86 | 0.65 | 0.67 | 0.77 | 0.79 | 0.58 | 0.59 | 0.87 | 0.90 |
|  | *0.89* | *0.93* | *0.68* | *0.74* | *0.75* | *0.74* | *0.78* | *0.88* | *0.89* | *0.93* |
| qc-W+B (M3, M7) | 0.65 | 0.68 | 0.69 | 0.72 | 0.84 | 0.88 | 0.71 | 0.77 | 0.88 | 0.95 |
|  | *0.64* | *0.86* | *0.75* | *0.83* | *0.85* | *0.88* | *0.70* | *0.91* | *0.92* | *0.98* |
| qc-W-B (M4, M8) | 0.84 | 0.87 | 0.85 | 0.88 | 0.91 | 0.96 | 0.82 | 0.87 | 0.90 | 0.95 |
|  | *0.84* | *0.95* | *0.86* | *0.76* | *0.87* | *0.81* | *0.80* | *0.91* | *0.91* | *0.93* |
| PSM_fb+B (M9, M13) | 0.69 | 0.69 | 0.68 | 0.72 | 0.70 | 0.72 | 0.71 | 0.74 | 0.92 | 0.95 |
|  | *0.80* | *0.91* | *0.72* | *0.81* | *0.66* | *0.74* | *0.79* | *0.95* | *0.92* | *0.91* |
| PSM_fb-B (M10, M14) | 0.88 | 0.89 | 0.78 | 0.81 | 0.82 | 0.85 | 0.84 | 0.87 | 0.94 | 0.97 |
|  | *0.92* | *0.98* | *0.79* | *0.81* | *0.79* | *0.86* | *0.87* | *0.93* | *0.95* | *0.95* |
| PSM_lp+B (M11, M15) | 0.61 | 0.62 | 0.68 | 0.70 | 0.81 | 0.84 | 0.69 | 0.72 | 0.91 | 0.96 |
|  | *0.67* | *0.79* | *0.79* | *0.81* | *0.85* | *0.88* | *0.75* | *0.83* | *0.92* | *1.00* |
| PSM_lp-B (M12, M16) | 0.85 | 0.88 | 0.85 | 0.88 | 0.86 | 0.88 | 0.87 | 0.91 | 0.94 | 0.99 |
|  | *0.84* | *0.91* | *0.86* | *0.81* | *0.84* | *0.81* | *0.86* | *0.95* | *0.94* | *1.00* |
| HASQI_lin_ (M17) | 0.68 | 0.68 | 0.44 | 0.40 | 0.66 | 0.66 | 0.62 | 0.61 | 0.82 | 0.85 |
|  | 0.65 | *0.64* | *0.44* | *0.48* | *0.56* | *0.62* | *0.58* | *0.52* | *0.79* | *0.74* |
| HASQI_nonlin_ (M18) | 0.49 | *0.49* | 0.51 | 0.52 | 0.76 | 0.79 | 0.76 | 0.78 | 0.79 | 0.84 |
|  | *0.23* | *0.17* | *0.52* | *0.55* | *0.85* | *0.95* | *0.83* | *0.74* | *0.81* | *0.90* |
| HASQI_comb_ (M19) | 0.54 | 0.54 | 0.50 | 0.51 | 0.78 | 0.79 | 0.73 | 0.76 | 0.81 | 0.85 |
|  | *0.24* | *0.19* | *0.53* | *0.62* | *0.86* | *0.95* | *0.80* | *0.69* | *0.87* | *0.88* |
| PESQ (M20) | 0.55 | 0.56 | 0.75 | 0.77 |  |  | 0.90 | 0.92 |  |  |
|  | *0.37* | *0.45* | *0.77* | *0.81* |  |  | *0.90* | *0.83* |  |  |
| PESQ-WB (M21) | 0.64 | 0.64 | 0.62 | 0.63 |  |  | 0.83 | 0.85 |  |  |
|  | *0.41* | *0.45* | *0.79* | *0.81* |  |  | *0.90* | *0.88* |  |  |
| LPD (M22) | -0.75 | -0.77 | -0.52 | -0.57 |  |  | -0.51 | -0.55 |  |  |
|  | *-0.64* | *-0.62* | *-0.37* | *-0.43* |  |  | *-0.53* | *-0.57* |  |  |
| *D* (M23) | -0.72 | -0.72 | -0.44 | -0.43 |  |  | -0.53 | -0.52 |  |  |
|  | *-0.63* | *-0.64* | *-0.41* | *-0.38* |  |  | *-0.57* | *-0.52* |  |  |
| *R_nonlin_* (M24) | *0.68* | *0.69* | *0.52* | *0.54* |  |  | 0.75 | *0.78* |  |  |
|  | *0.41* | *0.40* | *0.53* | *0.62* |  |  | *0.77* | *0.69* |  |  |
| *S_overall_* (M25) | 0.76 | 0.76 | 0.50 | 0.51 |  |  | 0.66 | 0.69 |  |  |
|  | *0.63* | *0.64* | *0.42* | *0.48* |  |  | *0.62* | *0.64* |  |  |
| ISD (M26) | -0.14 | -0.25 | -0.20 | -0.35 |  |  | -0.03 | -0.24 |  |  |
|  | *-0.49* | *-0.50* | *-0.26* | *-0.31* |  |  | *-0.18* | *-0.31* |  |  |
| LAR (M27) | -0.83 | -0.84 | -0.62 | -0.65 |  |  | -0.61 | -0.63 |  |  |
|  | *-0.74* | *-0.71* | *-0.49* | *-0.48* |  |  | *-0.65* | *-0.67* |  |  |
| LLR (M28) | -0.75 | -0.76 | -0.51 | -0.54 |  |  | -0.45 | -0.48 |  |  |
|  | *-0.78* | *-0.81* | *-0.50* | *-0.48* |  |  | *-0.65* | *-0.71* |  |  |
| WSSD (M29) | -0.67 | -0.70 | -0.52 | -0.54 |  |  | -0.78 | -0.81 |  |  |
|  | *-0.53* | *-0.64* | *-0.40* | *-0.52* |  |  | *-0.69* | *-0.79* |  |  |

Overall results (linear and rank correlation (*italic*) coefficients for all tested measures and subjects). Quality measure suffixes: fb/lp: modulation filterbank/lowpass model version; +/-W: with/without frequency band weighting; +/-B: with/without asymmetric weighting of differences ("Beerends weighting").
